# Cryptococcus neoformans Database in Synthetic Biology Open Language

**DOI:** 10.1128/mra.00198-22

**Published:** 2022-08-24

**Authors:** Sophia Garcia de Resende, Ana Carolina Campos Batista, Mayna da Silveira Gomide, Luis Henrique Scarparo Pandolfo, Leonardo Ferreira da Silva, Ildinete Silva-Pereira, Cíntia Marques Coelho

**Affiliations:** a Laboratory of Synthetic Biology, Department of Genetics and Morphology, Institute of Biological Science, University of Brasília (UnB), DF, Brazil; b Laboratory of Molecular Biology of Pathogenic Fungi, Department of Cellular Biology, Institute of Biological Science, University of Brasilia (UnB), Brasília, DF, Brazil; c School of Medicine, Federal University of Juiz de Fora (UFJF), Juiz de Fora, MG, Brazil; University of California, Riverside

## Abstract

Cryptococcus neoformans is the etiologic agent of cryptococcosis, a lethal worldwide disease. Synthetic biology could contribute to its better understanding through engineering genetic networks. However, its major challenge is the requirement of accessible genetic parts. The database presented here provides 23 biological parts for this organism in Synthetic Biology Open Language.

## ANNOUNCEMENT

Cryptococcus neoformans is a facultative intracellular basidiomycete fungus, which became an important human pathogen after the HIV/AIDS pandemic, mostly affecting immunocompromised patients (1–5). In 2014, the global incidence of cryptococcal meningitis was about 200,000 annually, resulting in around 180,000 annual deaths (6).

This medically important organism already had its genome sequenced (7), and several techniques that enable its genetic manipulation have also been developed (8). Even methods based on the recent gene editing system, CRISPR-Cas9, have been shown to be successful in C. neoformans (9–12). However, other synthetic biology tools, such as construction of genetic circuit networks that could advance the comprehension of its biology and pathogenicity, face constraints. One of the key points in this area is the standardization that supports description and characterization of the basic biological genetic parts such as promoters, terminators, and coding sequences (13). Recently, SynBioHub, a repository that allows researcher to exchange parts in Synthetic Biology Open Language (SBOL), was created (14), but those parts are not promptly available for nonmodel organisms.

Thus, the present study aimed to develop and make available to the scientific community an SBOL format database composed of successfully functionally evaluated genetic biological parts for C. neoformans.

**FIG 1:**
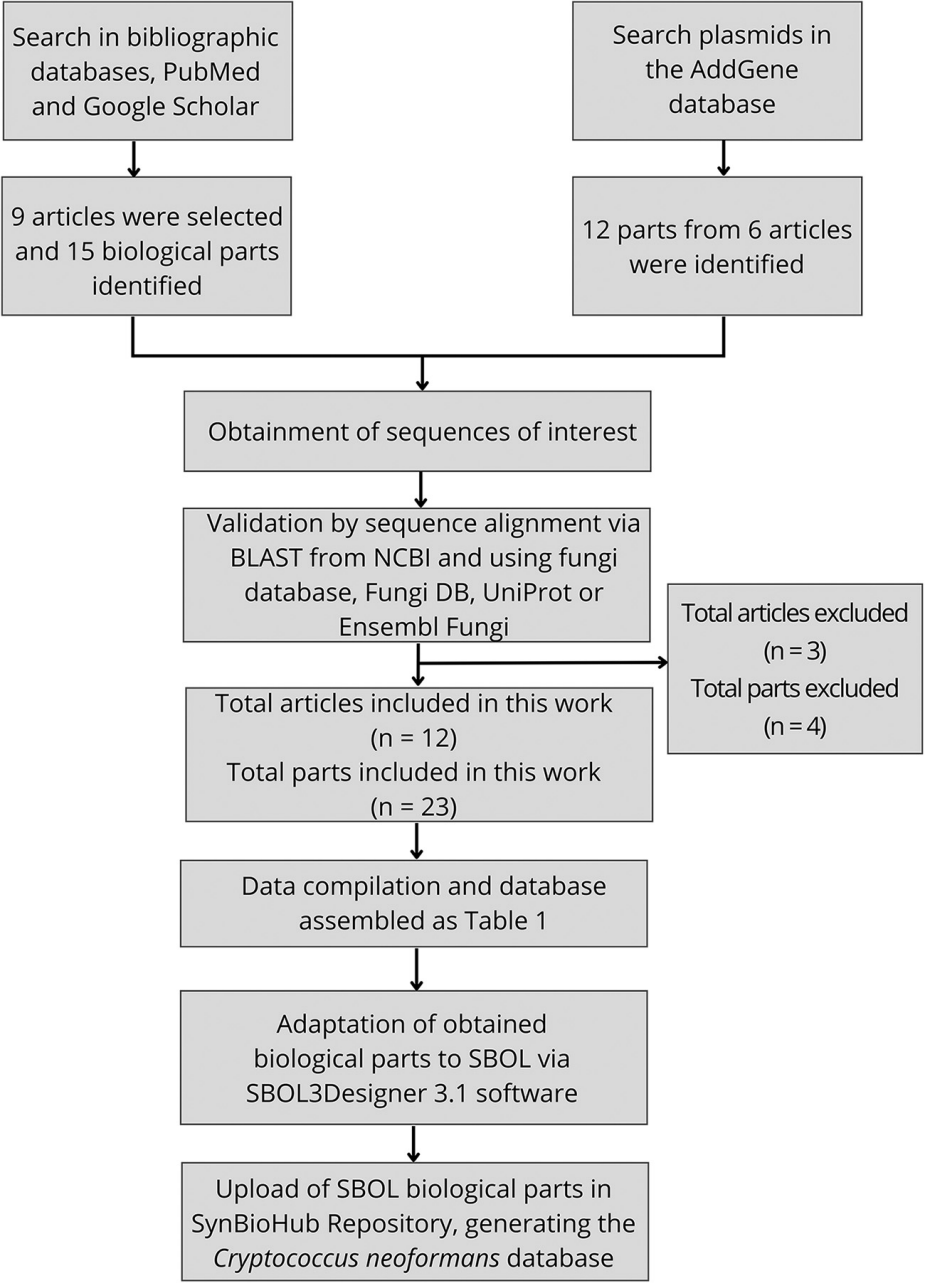
Schematic representation of the strategy followed for Cryptococcus neoformans database assembly. At first a comprehensive evaluation of the literature for genetic biological parts from C. neoformans was performed in different bibliographic databases, such as PubMed and Google Scholar, with the following terms: promoters, coding sequences, terminators, and the target organism. In addition, the Addgene database was searched with the same terms. Only the genetic biological parts that met specific criteria such as the sequence being complete, being expressed, and being used in the target organism were selected for the following step. BLAST alignments were performed to confirm the genetic part sequence, followed by a search on FungiDB, UniProt, or Ensembl Fungi. Twenty-three parts from 12 articles were included in the database, and four genetic parts were excluded for not complying with the required criteria. The data were compiled in a table (https://doi.org/10.6084/m9.figshare.19990838.v2). The synthetic biology design tool, SBOLDesigner3.1, was used to create and to provide standardized visualizations of these individual genetic parts in the Synthetic Biology Open Language (SBOL), which were then deposited in the SynBioHub repository.

Figure 1 describes the schematic strategy set to develop this study. Initially, a search in the literature was performed, using bibliographic databases such as PubMed and Google Scholar. The Biobricks terms used were promoters, coding sequences, terminators, and the organism’s name. From this, 9 articles were selected, and those were searched for whether the sequence was complete, whether the article contained the gene identifier (ID), or whether there was a genomic coordinate of the biological part. These 9 articles contained 15 parts, although 4 genetic parts from 3 articles were not admitted as they did not meet the verification criterion requirements, which were that the sequence is complete, it is functional, and it is in use in the target organism. The Addgene database was also searched using the same terms. There, 12 biological parts were found in 6 articles. In total, 12 articles for the 23 parts were accepted in this database, after a validation step through an alignment program, such as the Basic Local Alignment Search Tool, from NCBI followed by a search on FungiDB, UniProt, or Ensembl Fungi. Finally, these parts were deposited in SynBioHub, using SBOLDesigner3.1 (15, 16).

The table at figshare presents the information on each biological part that composes the C. neoformans database (https://doi.org/10.6084/m9.figshare.19990838.v2). The promoters were acquired from genes encoding actin (ACT1) (9), antiphagocytic protein (App1) (17), galactose kinase (GAL1) (18), galactose transferase (GAL7) (18, 19), UDP-glucose epimerase (UGE2) (18), glyceraldehyde-3-phosphate dehydrogenase (GPD) (20), U6 (CnU6) (10), orotidine monophosphate pyrophosphorylase (URA5) (21), and elongation factor 1 (TEF1) (9). The coding sequences were acetamidase (AmdS) (22), Cas9 (9, 23), the fluorescent protein mCherry (24), green fluorescent protein (GFP) (24), URA5 (21), hygromycin (HYG) (22, 25), neomycin (NEO/G418) (22, 26), and nourseothricin (NAT/NrsR) (24, 27) resistance genes. The terminators belonged to genes encoding phosphoribosylanthranilate isomerase (TRP1) (22), HOG1 (HOG1t) (24), TEF1 (9), CnU6 (10), and URA5 (21). In conclusion, the database is ready and accessible to all the community in the Synthetic Biology area.

### Data availability.

Detailed information on the Cryptococcus neoformans database can be found at https://doi.org/10.6084/m9.figshare.19990838.v2 and in Synthetic Biology Open Language, which is available and can be accessed in SynBioHub by the following link: https://synbiohub.org/public/CnDatabase/CnDatabase_collection/1.
